# Evaluating antenatal breastmilk expression outcomes: a scoping review

**DOI:** 10.1186/s13006-021-00371-7

**Published:** 2021-03-12

**Authors:** Imane Foudil-Bey, Malia S. Q. Murphy, Sandra Dunn, Erin J. Keely, Darine El-Chaâr

**Affiliations:** 1 OMNI Research Group, Clinical Epidemiology Program, Ottawa Hospital Research Institute, Ottawa, Canada; 2 Faculty of Medicine, University of Ottawa, Ottawa, Canada; 3 Division of Endocrinology and Metabolism, Department of Medicine, University of Ottawa, Ottawa, Canada; 4 Diabetes, Obesity, Metabolism and Endocrinology Program, Ottawa Hospital Research Institute, Ottawa, Canada; 5 Foustanellas Endocrine & Diabetes Centre, The Ottawa Hospital, Ottawa, Canada; 6 Department of Obstetrics and Gynecology, University of Ottawa, Ottawa, Canada; 7 Department of Obstetrics, Gynecology & Newborn Care, The Ottawa Hospital, General Campus, CPCR, Box 241, 501 Smyth Rd, Ottawa, ON K1H 8L6 Canada

**Keywords:** Pregnancy, Colostrum, Antenatal breastmilk expression, Breastfeeding, Antenatal breast expression

## Abstract

**Background:**

Antenatal breastmilk expression (aBME) is recommended by some healthcare providers to improve lactation, breastfeeding, and newborn outcomes, particularly for women with diabetes as they face unique challenges with breastfeeding. However, there is limited evidence of the potential harms and benefits of this practice. Our objective was to conduct a scoping review to map the literature describing maternal and newborn outcomes of aBME.

**Methods:**

We searched Medline, Embase, CINAHL, Cochrane Database of Systematic Reviews, British Library E-Theses Online Services (EThOS) database, OpenGrey, and Clinical trials.gov from inception to January 2020. Studies in English that reported on the effect of aBME on maternal and newborn outcomes, and the experiences of women who have engaged in the practice were included for screening. Titles, abstracts, and full-text articles were screened by two independent reviewers. A critical appraisal and clinical consultation were conducted. Key findings were extracted and summarized.

**Results:**

We screened 659 studies and 20 met the inclusion criteria. The majority of included studies (*n* = 11, 55.0%) were published after 2015, and seven (35.0%) originated from Australia. Ten (50.0%) studies provided data on high-risk obstetrical populations, including those with diabetes (*n* = 8), overweight or obesity (*n* = 1), and preeclampsia (*n* = 1). Commonly reported outcomes included breastfeeding status at discharge or follow-up, mode of delivery, newborn blood glucose, and time to establishing full lactation. Maternal experiences were related to challenges with aBME, confidence and mastery, perceived impact, security and altruism, learning and resources, and physical symptoms as a result of aBME. The critical appraisal demonstrated limited high-quality evidence surrounding aBME.

**Conclusions:**

Our findings demonstrate increasing interest in the safety, efficacy, and acceptability of aBME. Existing studies are heterogenous with variable research questions, outcomes, study designs, and methodology. The recommendations made in this review can be used to help inform  future studies evaluating aBME.

**Supplementary Information:**

The online version contains supplementary material available at 10.1186/s13006-021-00371-7.

## Background

The World Health Organization, Public Health Agency of Canada, Health Canada, Canadian Pediatric Society, and the Breastfeeding Committee for Canada recommend exclusive breastfeeding to 6 months of age and up to 2 years of life and beyond with appropriate introduction of solid foods [1–5]**.** Exclusive breastfeeding is defined as the receipt of solely human milk (infant breastfeeds or receives expressed or donor milk), as well as oral rehydration solutions and syrups (vitamins, minerals, medicine) if needed [6]. Clinical practice guidelines recommend that breastfeeding be initiated as soon as possible after birth as it provides many well accepted benefits to infant and maternal health [7–10].

Initiation of breastfeeding in hospital can be challenging in situations where the normal process of lactogenesis (milk production) or milk transfer to the newborn is delayed or impeded. Most women experience copious milk production 2–3 days postpartum (lactogenesis II) [8]; however, women with diabetes in pregnancy have been shown to experience a delay or absence of this process [8, 9]. Newborns of mothers with higher risk pregnancies (i.e. diabetes, hypertension, epilepsy, or other chronic diseases in pregnancy) have an increased risk of developing neonatal complications requiring admission to the neonatal intensive care unit (NICU). This may lead to prolonged separation of the newborn from its mother which may also impede breastfeeding initiation. Additionally, newborns of mothers with diabetes are at risk of hypoglycemia directly after birth and often require infant formula or intravenous glucose [7], which may interfere with breastfeeding initiation and consequent maintenance [11]. In such cases, the use of breastmilk may be needed to supplement feeding, especially since colostrum has been shown to be more effective at stabilizing blood glucose than infant formula [12]. In scenarios where the production of breastmilk immediately after birth may be difficult, antenatal breastmilk expression (aBME) may be a feasible intervention to encourage storage of colostrum for postpartum use [13–15].

Antenatal breastmilk expression (aBME) has demonstrated potential to hasten lactogenesis II [16], decrease postpartum breast engorgement, avoid the need for breastmilk substitutes (infant formula), improve blood glucose stabilization in newborns at risk of hypoglycemia [15, 17, 18], and increase exclusive breastfeeding maintenance up to 6 months postpartum [10]. However, the association between antenatal breast stimulation and oxytocin release raises concern about the possible induction of preterm labour or miscarriage [15, 19, 20]. Findings from observational studies suggest that mothers with diabetes who engage in aBME may be at increased risk of preterm labour and neonatal admission to the NICU [13, 20]. In 2017, Forster et al. published the first randomized control trial (RCT) evaluating the safety and efficacy of aBME in mothers with diabetes [21]. Based on their primary outcome of newborn admission to the NICU, the trial did not report any evidence suggesting that aBME is harmful in low risk women with diabetes [21]. Although aBME is widely implemented as part of lactational and maternity support programs [22–24], evidence evaluating its safety and efficacy has largely stemmed from small observational studies with methodological limitations. A synthesis of the literature is warranted to inform clinical decision-making and future research.

Our objective was to conduct a scoping review to summarize and appraise previous approaches to evaluating maternal and newborn outcomes associated with aBME. The scoping review methodology was suitable as it allowed the authors to include multiple study designs and explore a broad clinical question [25].

## Methods

### Sources

This review was registered on Open Science Framework [26] and the protocol has been described in detail elsewhere [27]. Research ethics board approval was not required due to the nature of the study methodology. Our methodology was developed using a multi-step approach proposed by the Joanna Briggs Institute [28] in conjunction with the seminal scoping review frameworks [29, 30]. Our approach included: identification of the research question, identification of relevant studies, selection of studies, charting of data, critical appraisal, summary of results, and clinical consultation.

The development of our search strategy was an iterative process and was conducted in consultation with a medical librarian. The finalized search strategy is provided in the Additional file 1. Our search criteria were applied to the following electronic databases: Medline (OVID), Embase (OVID), CINAHL (EBSCOHost), Cochrane Database of Systematic Reviews (OVID), British Library E-Theses Online Services (EThOS) database, and OpenGrey. Clinical trials.gov was searched for any ongoing trials. We ran our first search on June 26, 2019 and ran a second search in January 2020 to ensure that studies published up to December 31, 2019 were captured. Records were exported from each database and uploaded to the Covidence web-based software platform [31]. Thereafter, duplicate citations were removed.

### Study selection

Eligible studies included primary research on pregnant women (population) that evaluated maternal (including maternal experiences and perspectives, maternal health outcomes, aBME outcomes, and breastfeeding outcomes) or newborn outcomes (concept) following aBME (context). Due to the anticipated small number of studies on this topic, no limits were placed on the geography, environment, or timeframe. Studies unavailable in English, or for which the full-text articles could not be retrieved were excluded.

Title, abstract, and full text screening were conducted by two independent reviewers (IFB and MSQM). If titles and abstracts met the minimum inclusion criteria, the corresponding studies proceeded to full text review. Full texts were imported and screened in further detail for eligibility by the two reviewers. When consensus was not reached, a third, independent reviewer was consulted (DEC). The reference lists of included articles were screened for any publications that were missed by the electronic database search. The reference lists of secondary literature that were not included in our study but were retrieved from our search strategy were also screened to identify primary studies that may have been missed.

Key concepts and bibliometric data were independently extracted from all included articles by the two independent reviewers (IFB and MSQM) using a data collection form designed by the two reviewers. A template of the data collection form is provided in the Additional file 2. Although we include our assessment of all studies captured by our search strategy, we caution readers regarding the interpretation of the results of historical studies, such as those published in the 1940s and 1950s, as the research questions, study designs and study interventions are notably different than those of more recent investigations.

A critical appraisal was conducted by two independent reviewers (MSQM and SD) using the Mixed Methods Appraisal Tool (MMAT) – Version 2018 [32]. The MMAT is designed to assess the validity and strength of five study types: qualitative research, RCTs, non-randomized studies, quantitative descriptive studies, and mixed methods studies. Articles are assessed based on reviewer responses to five questions specific to each study type. Responding with ‘no’ or ‘can’t tell’ to any of the rating criteria for a given study design indicates that the study does not report appropriate information related to the criterion, or that the presented information is unclear. An overall score for each article is not given; rather, authors provided comments to support their ratings for each criterion. For the purpose of summarizing the results of the critical appraisal, we have provided numerical values that represent the number of ‘yes’ responses for each included article.

A clinical expert (EJK) was consulted to identify potential additional sources of information and provide further insight into the clinical applicability of the scoping review. The clinical expert provided unique perspectives using their clinical experience that guided the discussion. The clinical consultation allowed the authors to share preliminary data, guided interpretation of the results, and identified appropriate knowledge translation and dissemination strategies [30].

## Results

**Fig. 1:**
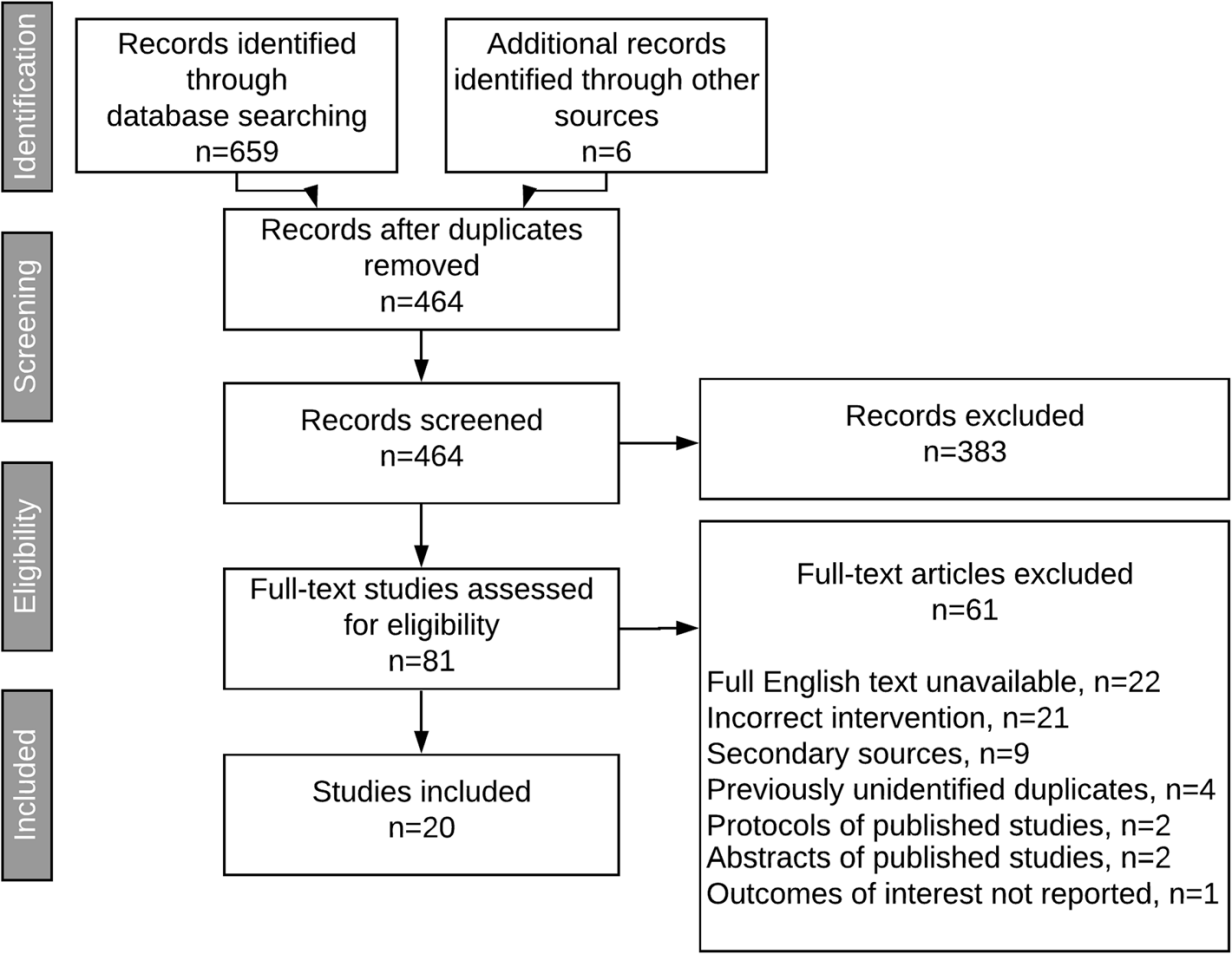
PRISMA flow diagram

A total of 659 studies were identified by our search strategy. One hand-picked study that was not identified by our search was included, and five were identified from the reference lists of included studies. Following removal of 201 duplicates, 464 records remained for title and abstract screening. Three hundred eighty-three titles and abstracts did not meet the minimum inclusion criteria and 81 proceeded to full-text screening. Upon full-text review, 61 articles were excluded due to unavailability of the full-text, incorrect intervention, incorrect source of data, duplicate, or reporting on outcomes unrelated to aBME. A total of 20 full-text articles met the inclusion criteria and were therefore included in the scoping review. A summary of the titles, abstracts, and full texts reviewed in the course of this review is provided in Fig. 1.

Included studies were published between 1946 and 2019. Eleven (55.0%) were published within the last 5 years (2015–2019) [21, 24, 33–41]. Included studies originated from six countries: Australia (*n* = 7) [13, 21, 24, 33–36], New Zealand (*n =* 1) [42], the United Kingdom (*n* = 5) [20, 39, 43–45], Sweden (*n* = 1) [46], United States (*n* = 3) [37, 38, 47], and India (*n* = 3) [16, 40, 41]. Three dominant research groups were identified; Forster et al. [13, 21], Demirci et al. [37, 38], and Casey et al. [35, 36], have each published two studies included in our review. Of note, the three independent studies published in India had very similar sample sizes, study objectives, and interventions [16, 40, 41].

A detailed summary of the studies included in this scoping review is provided in Table 1. This review included one quality improvement study [33], two case studies/series [38, 43], four qualitative and/or cross sectional survey studies of the knowledge, attitudes and experiences of women engaging in aBME [34, 35, 37, 39], three observational cohort studies [20, 36, 46], and ten interventional studies [13, 16, 21, 24, 40–42, 44, 45, 47] including one RCT [21]. Sample sizes of included studies ranged from 1 to 60 (*n* = 8) [13, 34, 35, 37, 38, 42, 43, 47], 80–230 (*n* = 8) [16, 20, 24, 33, 40, 41, 44, 45], and 300–690 (*n* = 4) [21, 36, 39, 46] participants.

**Table 1:** Study characteristics (*n* = 20)

Author (year of publication), Country of origin	Study objective(s)	Study design	Study setting	Sample size	Study population	Timing of aBME
Blaikley et al. (1953) [44] United Kingdom	Breast-feeding: factors affecting success A report of a trial of the Woolwich methods in a group of primiparae.	Interventional study	Hospital lying-in ward	*n* = 222	Pregnant women	After 32–33 weeks’ gestation
Brisbane et al. (2015) [34] Australia	To discuss the qualitative outcomes of women who attended a prenatal care clinic at 37 weeks’ gestation which supports the antenatal expression of colostrum.	Descriptive study - Qualitative study and/or survey	Antenatal breastfeeding clinic	*n* = 57	Pregnant women	After 37 weeks’ gestation
Brown et al. (1975) [47] United States	Preparation of the breast for breastfeeding.	Interventional study – randomized prospective cohort	Hospital in Colorado	*n* = 57	Pregnant women	3 weeks prior to expected delivery
Casey et al. (2019) [36] Australia	To compare rates of neonatal hypoglycaemia in babies born to mothers who express and store antenatal colostrum to babies born to mothers who do not.	Observational study - Retrospective cohort	Regional public hospital	*n* = 303	Pregnant women with diabetes	Between 34 and 36 weeks’ gestation
Casey et al. (2019) [35] Australia	To explore the perspectives and experiences of women who have had diabetes in pregnancy and were encouraged to collect and store colostrum in the antenatal period.	Descriptive study - Qualitative study and/or survey	Regional public hospital	*n* = 6	Pregnant women with diabetes	Not defined
Clay (2005) [43] United Kingdom	To provide an example of a collaborative partnership approach between a multidisciplinary team in a joint antenatal diabetes clinic and a mother with type 1 diabetes to help her experience the harvesting of colostrum in the antenatal period and enable a positive breast-feeding outcome for her newborn daughter.	Descriptive study - Case study	Antenatal diabetes clinic	*n* = 1	Pregnant woman with type 1 diabetes	After 36 weeks’ gestation
Demirci et al. (2018) [38] United States	To report on maternal experiences and breastfeeding outcomes in mothers with a hypertensive disorder of pregnancy who engaged in aBME.	Descriptive study - Case series	Hospital-based midwife practice	*n* = 4	Pregnant women with hypertension	Beginning ‘around’ 37 weeks’ gestation
Demirci et al. (2019) [37] United States	To examine the experiences of first-time mothers in the United States who participated in a pilot study of aBME.	Descriptive study - Qualitative study and/or survey	Hospital-based midwife practice	*n* = 19	Pregnant women	After 37 weeks’ gestation
Fair et al. (2018) [39] United Kingdom	To assess women’s knowledge, practices, and opinions of aBME as well as any differences within the overweight and obese subgroups.	Descriptive study - Qualitative study and/or survey	Online recruitment through a maternity service user and Facebook parenting group	*n* = 688	Women who are pregnant or who have given birth	Between 34 and 36 weeks' gestation
Forster et al. (2011) [13] Australia	To determine the feasibility and begin assessing the safety and efficacy of conducting a randomised control trial to evaluate antenatal breastmilk expression in mothers with diabetes.	Interventional study - Pilot study	Public, tertiary, women’s hospital	*n* = 43	Pregnant women with diabetes	After 36 weeks’ gestation
Forster et al. (2017) [21] Australia	To determine the safety and efficacy of aBME in women with diabetes in pregnancy.	Interventional study - Randomised controlled trial	Multi-center study across six hospitals	*n* = 632	Pregnant women with diabetes	After 36 weeks’ gestation
Ingelman-Sundberg (1958) [46] Sweden	To study the advantages of antenatal nipple message and expression of colostrum in pregnant women.	Observational study - Prospective cohort	Private lying-in ward	*n* = 656	Pregnant women	After 20 weeks’ gestation
Lamba et al. (2016) [40] India	To study the effect of aBME at term pregnancy and subsequent effect on postnatal lactation performance.	Interventional study	Tertiary care hospital	*n* = 200	Pregnant women	After 37 weeks’ gestation
O’Sullivan et al. (2019) [24] Australia	To determine whether an online instructional video can improve knowledge and confidence around the antenatal expression of colostrum.	Interventional study - before/after comparison	Online recruitment through social media via university and research institutions, infant and mother organizations, and personal contacts	*n* = 95	Pregnant women	Not defined
Rietveld (2011) [42] New Zealand	To determine if pregnant woman with Type 1, Type 2 or gestational diabetes mellitus can effectively achieve antenatal colostrum harvesting and banking. To assess the feasibility of mothers and core midwifery staff using banked colostrum as part of the care of hypoglycaemic babies in the hospital setting.	Interventional study - Pilot study	Antenatal diabetic outpatient clinic	*n* = 10	Pregnant women with diabetes	After 34 weeks’ gestation
Singh et al. (2009) [16] India	To study the effect of aBME at term in reducing breast feeding failure compared to conventional method of breastfeeding initiation.	Interventional study	Not stated	*n* = 180	Pregnant women	After 37 weeks’ gestation
Soltani et al. (2012) [20] United Kingdom	To examine the adoption of aBME as an intervention and investigate its relationship to birth outcomes among mothers with diabetes.	Observational study - Retrospective cohort	National health service trust	*n* = 85	Pregnant women with diabetes	After 36 weeks’ gestation
Uikey et al. (2017) [41] India	To study the effect of aBME in improving lactational performance.	Interventional study	Tertiary care center	*n* = 200	Pregnant women	After 37 weeks’ gestation
Waller (1946) [45] United Kingdom	The early failure of breastfeeding: A clinical study of its cause and their prevention.	Interventional study	Hospital lying-in ward	*n* = 200	Pregnant women	Last 3 months of pregnancy
Weinel et al. (2019) [33] Australia	To support mothers who had pre-existing or gestational diabetes, and who were on more than 20 units of insulin per day, to express colostrum in the antenatal period at 36 weeks’ gestation and to promote breastfeeding.	Quality improvement study	Diabetic antenatal care education clinic	*n* = 207	Pregnant women with diabetes	After 36 weeks’ gestation

Whereas ten (50.0%) studies examined aBME practices in the general obstetrical population [16, 24, 34, 37, 40, 41, 44–47], and ten studies (50.0%) reported on maternal or newborn outcomes as they related to high-risk populations including women with diabetes (*n* = 8) [13, 20, 21, 33, 35, 36, 42, 43], hypertensive disorders of pregnancy (*n* = 1) [38], and overweight or obesity (*n* = 1) [39].

Study settings and data sources included hospitals/tertiary care centers (*n* = 10) [13, 20, 21, 35–38, 40, 41, 47], outpatient clinics (*n* = 4) [33, 34, 42, 43], online surveys (*n* = 2) [24, 39], and private ‘lying in’ wards (*n* = 3) [44–46]. One study did not provide the details of the study setting [16].

The timing of aBME varied across studies, with the majority of participants expressing at, or after, 37 (*n* = 6) [16, 34, 37, 38, 40, 41] or 36 (*n* = 5) [13, 20, 21, 33, 43] weeks’ gestation. Less frequently, studies included women expressing at, or after, 20 (*n* = 1) weeks’ gestation [46], or between 32 and 36 (*n* = 4) [36, 39, 42, 44] weeks’ gestation. Brown et al. implemented aBME 3 weeks prior to each mother’s expected date of delivery [47], and Waller, published in 1946, recommended the intervention in the last 3 months of pregnancy [45]. Two studies did not specify the timing of aBME [24, 35].

**Fig. 2:**
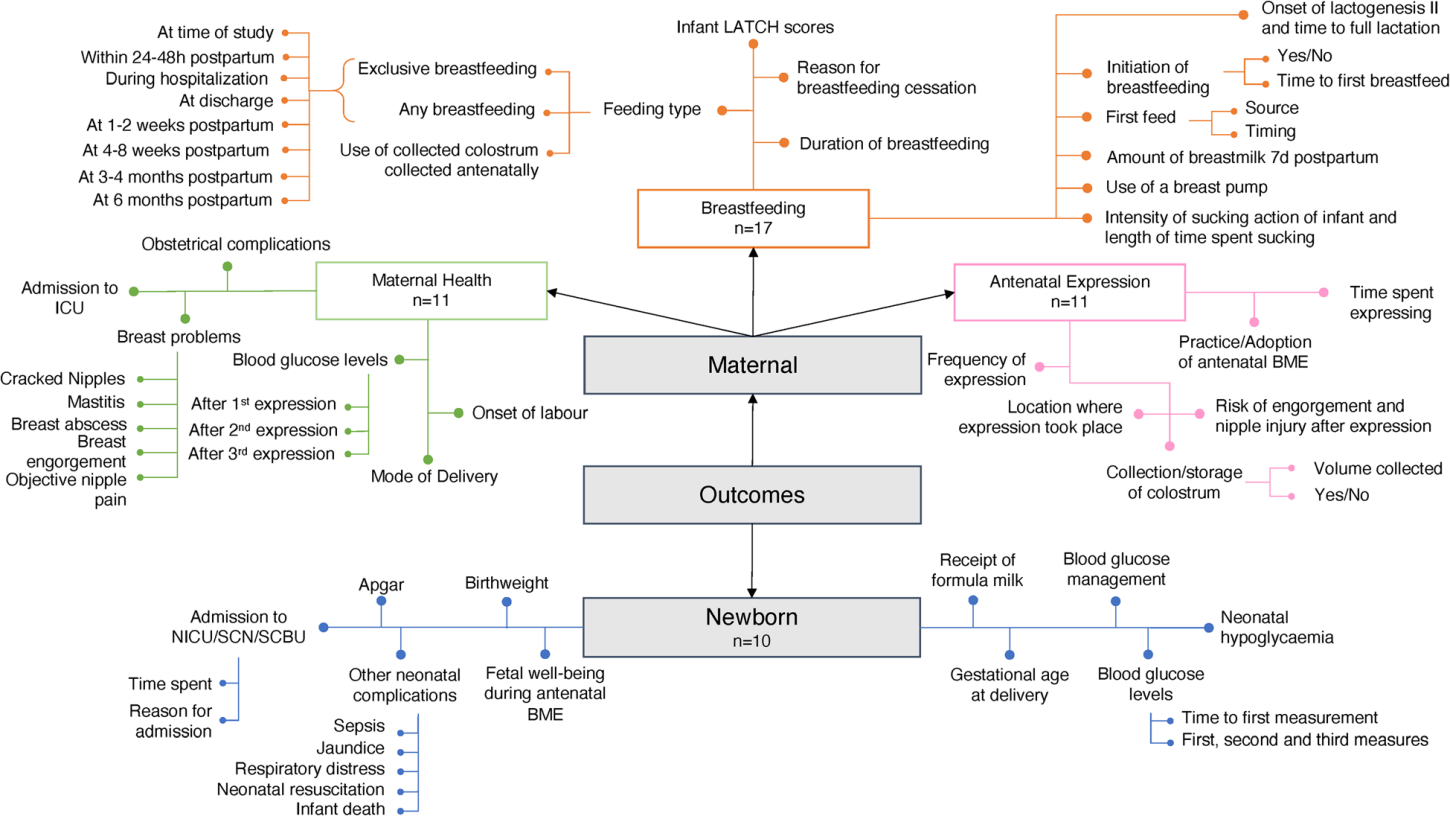
Conceptual map of reported maternal and newborn outcomes in included studies (*n* = 20). The number of studies reporting on each given outcome category is provided

A concept map of the maternal and newborn outcomes assessed in the included studies is provided in Fig. 2. Maternal outcomes were categorized by those specific to the aBME practice, those pertaining to breastfeeding, and those related to downstream maternal health.

Eleven studies (55.0%) collected data directly related to the aBME practice [13, 21, 24, 34, 36, 37, 39, 42, 43, 45, 47]. These studies reported on the adoption of aBME [34, 39], prior knowledge of aBME [24, 39], the setting and time of implementation [13], the duration and frequency of expressing episodes [13, 21, 37, 47], and the collection of colostrum (including volume collected and use of colostrum in hospital) [13, 21, 34, 36, 37, 42, 43].

Eleven studies (55.0%) reported on maternal health outcomes among women who practiced aBME [13, 20, 21, 37, 38, 40, 41, 43, 44, 46, 47]. These outcomes included maternal blood glucose levels after aBME episodes [13, 21, 43], obstetrical complications [37], risk of engorgement and nipple injury with antenatal expression [45], onset of labour [20, 21], mode of delivery [20, 21, 37, 40, 41], breast problems during breastfeeding (cracked nipples, mastitis, formation of a breast abscess, breast engorgement, nipple pain) [20, 44, 46, 47], and postpartum maternal admission to the ICU [38].

Seventeen studies (85.0%) provided data on breastfeeding outcomes which ranged from those immediately after delivery to those at 6 months postpartum [13, 16, 20, 21, 33, 34, 36–38, 40–47]. Breastfeeding outcomes were primarily related to newborn feeding sources (breastmilk and/or infant formula supplementation) at various times postpartum. Reported breastfeeding outcomes included perceived timing of onset of lactogenesis II [21, 38, 43], time to full lactation [16, 40, 41], initiation of breastfeeding and duration [20, 34, 41], source of and time to first feed [38, 42], receipt of infant formula and/or breastmilk at various time points [13, 21, 36, 44, 45], feeding type in hospital including the use of antenatally expressed milk [38], newborn LATCH scores [38], breastfeeding status at various time points postpartum [33, 37, 38, 42, 44, 46], use of an electric pump [37], and reasons for breastfeeding cessation [34]. One study measured the total amount of breastmilk suckled and expressed 7 days after delivery [46], and another reported on the intensity of sucking action of the newborn, the number of minutes newborn sucked at each breast, and the length of time since the last feed [47].

Ten studies (50.0%) collected data on fetuses or newborns of mothers who practiced aBME [13, 20, 21, 36–38, 42–44, 47]. Assessed outcomes included metrics of fetal wellbeing during aBME [21], gestational age at birth [20, 21, 36, 37, 42], birthweight [20, 21, 37, 47], Apgar scores [20, 21, 36], blood glucose measurements (time to first measure, glucose levels at various time points) [13, 36], neonatal hypoglycemia [36, 42, 43], whether the newborn received intravenous glucose [13, 36], admission to NICU/SCN/SCBU (reason for admission, time spent) [13, 20, 21, 36–38], receipt of infant formula (various time points) [36–38, 42, 43], and other neonatal complications (jaundice, respiratory distress, sepsis, neonatal resuscitation, newborn death) [36, 37, 44].

Among all reported outcomes, the most frequently reported were breastfeeding status (at time of study, in first 48 h of life, at discharge, at 1–2 weeks, 4–8 weeks, 3 months, 4 months, and 6 months postpartum), mode of delivery (vaginal/cesarean), volume of colostrum collected, and newborn blood glucose measurements (time to first measure, glucose levels at 3, 12, and 24 h postpartum, or at the first, second, and third measurement).

**Fig. 3:**
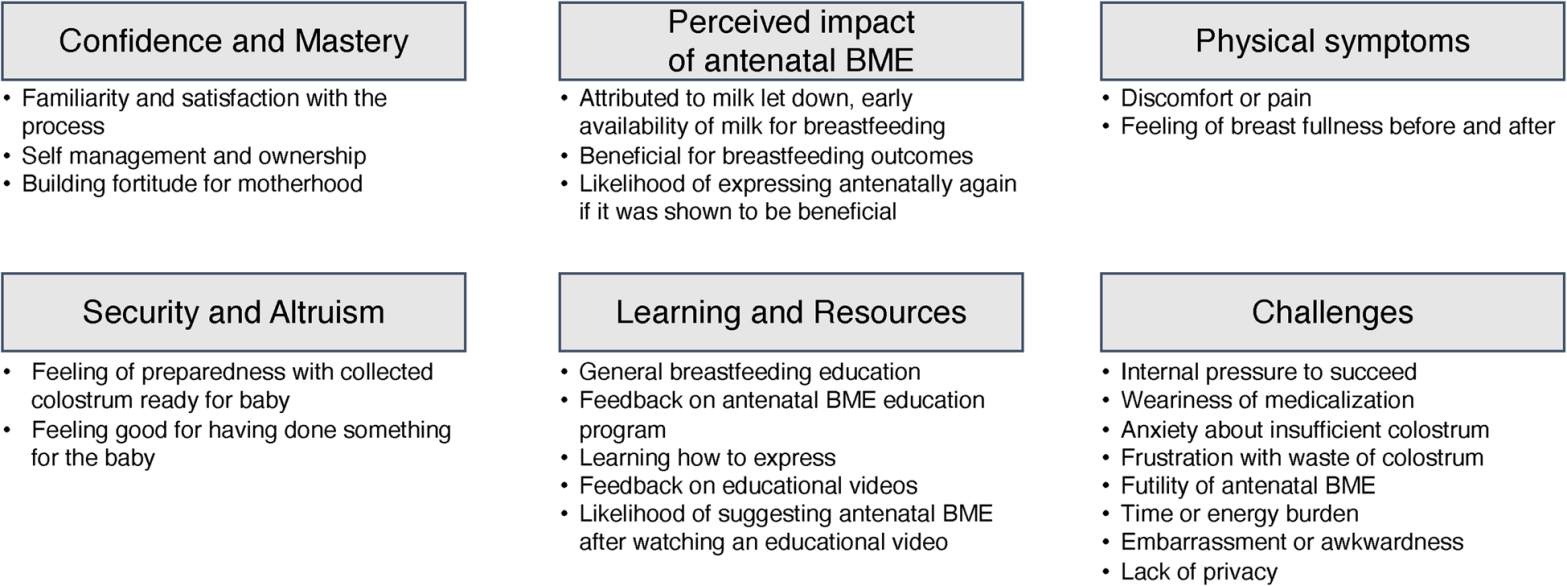
Maternal experiences grouped thematically based on recurrent reported outcomes (*n* = 10)

Ten studies (50.0%) provided evidence on maternal knowledge, attitudes, and experiences related to aBME [13, 24, 34, 35, 37–40, 42, 47]. Data were obtained through patient diary documentation [13], structured or semi-structured postpartum interviews [34, 35, 37, 38], questionnaires [13, 24, 34, 39, 47], and online surveys (fixed and free text responses) [38, 42]. One study did not specify how they obtained data on maternal experiences [40]. Broadly categorized by theme, these studies collected information regarding participants’ sense of confidence and mastery as a result of aBME; senses of security and altruism attributed to aBME; receptivity to aBME resources or learning how to express; perceived impacts of aBME on maternal and newborn health; challenges related to attempting or practicing aBME; and the physical symptoms experienced as a consequence of aBME. Themes specific to maternal knowledge, attitudes, and experiences captured by included studies are summarized in Fig. 3.

The results of our critical appraisal are summarized in Table 2. Three studies (15%) did not meet the screening question criteria, indicating that they may not be empirical studies that could robustly be evaluated by the MMAT [33, 43, 46]. Five studies (25%) met 1/2 of the screening criteria questions [13, 38, 44, 45, 47], and 12 studies (60%) fulfilled both screening question criteria [16, 20, 21, 24, 34–37, 39–42]. Significant methodological weaknesses were evident in the majority of the studies reviewed. On average, the included studies successfully fulfilled 2.15/5 of the MMAT methodology appraisal criteria. Thirty-five percent (*n* = 7) fulfilled > 2/5 methodological appraisal questions [20, 21, 34, 35, 37–39]; four were qualitative studies [41, 42, 44, 45], one was a quantitative descriptive study [38], one was a quantitative RCT [21], and one was a quantitative non-randomized study [20]. Additionally, six of these seven studies (85.7%) were published in the last 5 years (2015–2019) [21, 34, 35, 37–39]. Only two studies fulfilled all five of the methodological quality appraisal criteria, both of which are qualitative in nature [35, 37]. Although there was only one registered RCT among the included studies [21], four other studies were evaluated as quantitative RCTs due to mention of a randomization process described in their methodology [16, 40, 41, 47]. A complete summary of all screening and methodology questions, results, and comments supporting the authors’ decisions are provided in the Additional file 3.

**Table 2:** Summary of Mixed Methods Appraisal Tool (MMAT) [32] results evaluating quality of included studies (*n* = 20)

Author (year of publication)	Screening questions (number of ‘yes’)	MMAT study design	Methodological quality questions (number of ‘yes’)
Blaikley et al. (1953) [44]	1/2	Quantitative non-randomized	2/5
Brisbane et al. (2015) [34]	2/2	Qualitative study	3/5
Brown et al. (1975) [47]	1/2	Quantitative randomized control trial	2/5
Casey et al. (2019) [36]	2/2	Quantitative non-randomized	2/5
Casey et al. (2019) [35]	2/2	Qualitative study	5/5
Clay (2005) [43]	0/2	Qualitative study	0/5
Demirci et al. (2018) [38]	1/2	Quantitative descriptive	4/5
Demirci et al. (2019) [37]	2/2	Qualitative study	5/5
Fair et al. (2018) [39]	2/2	Qualitative study	3/5
Forster et al. (2011) [13]	1/2	Quantitative non-randomized	2/5
Forster et al. (2017) [21]	2/2	Quantitative randomized control trial	3/5
Ingelman-Sundberg (1958) [46]	0/2	Quantitative non-randomized	0/5
Lamba et al. (2016) [40]	2/2	Quantitative randomized controlled trial	1/5
O’Sullivan et al. (2019) [24]	2/2	Qualitative study	2/5
Rietveld (2011) [42]	2/2	Mixed methods	2/5
Singh et al. (2009) [16]	2/2	Quantitative randomized control trial	1/5
Soltani et al. (2012) [20]	2/2	Quantitative non-randomized	3/5
Uikey et al. (2017) [41]	2/2	Quantitative randomized controlled trial	1/5
Waller (1946) [45]	1/2	Quantitative non-randomized	1/5
Weinel et al. (2019) [33]	0/2	Quantitative descriptive	1/5

Studies were commonly limited by unclear research questions or unclear descriptions of their intervention/outcome measures. Consequently, in many cases, we were unable to confirm whether findings appropriately addressed the indicated research question and were unable to effectively compare the findings across studies. A summary of methodological limitations identified is provided in Table 3.

**Table 3:** Common methodological flaws of included studies (*n* = 20)

• Research question or study outcomes related to aBME not well-defined	
• Incomplete description of study procedures preventing reproducibility (target population, inclusion/exclusion criteria, exposures/interventions, outcomes)	
• Limited description of the aBME protocol and supports provided to study participants	
• Limited information on the collection of colostrum from aBME, its use postpartum, and impact on the study outcomes	
• Inadequate or incomplete measures of lactational and breastfeeding outcomes	
• Limited information on participant retention and loss-to-follow-up, and comparative data between the final study sample and non-respondents/those lost during the course of the study	
• Lack of comprehensive baseline data on study participants	
• Limited collection or presentation of data on patterns of exclusive and mixed breastfeeding, pumping and infant formula supplementation, and little information on the reasons for infant formula supplementation (in-hospital or after discharge)	
• For randomized trials, limited description of randomization, allocation and blinding procedures	
• For quantitative studies, lack of pre-sample size calculations or power considerations	
• For quantitative studies, inappropriate matching of comparisons groups, or insufficient description of the matching criteria	
• For quantitative studies, not accounting for, or reporting on, participant compliance to the study intervention	
• For qualitative studies, limited information on the validity of the data collection tools, theoretical frameworks applied to guide study design or data analysis	
• For mixed methods studies, rationale for using a mixed-methods design was infrequently provided and there was limited integration of qualitative and quantitative findings	
• Not accounting for confounding variables or mediating factors (e.g. parity, obesity, breastfeeding history in previous pregnancies, socioeconomic status and breastfeeding supports received)	

## Discussion

Our findings demonstrate a rising interest in the safety, efficacy, and acceptability of aBME. Of the 20 studies identified by our search strategy, the majority were published in the last 5 years and originated from Australia. The current evidence surrounding aBME includes largely observational studies with low-risk mothers who were encouraged to express around 36 weeks’ gestation. Research questions, interventions, and outcomes of interest varied widely; however, breastfeeding outcomes were high yield (*n* = 17). A critical appraisal of included studies highlighted limitations in participant sampling, data collection, and reporting, suggesting research is warranted to rule out potential sources of bias and validate results. We identified only one RCT evaluating outcomes of aBME [21], however Demirci and colleagues registered a RCT in February of 2020 that will be assessing the effect of aBME on multiple breastfeeding outcomes in non-diabetic mothers with a body mass index > 25 kg/m^2^ [48].

Antenatal BME may be recommended to pregnant women for a wide range of reasons, including to promote lactogenesis, enhance breastfeeding success, and/or support collection of colostrum for postpartum use [13–15]. This was reflected in the studies included in this scoping review where the research questions were varied, instructions on the timing and method of aBME were inconsistent, and the outcomes of interest were often ambiguous. All of the studies that provided details on the method of breastmilk expression indicated hand expression, however the specific manner in which milk was expressed (i.e. nipple massage, nipple rolling, breast massage) varied and was often not detailed. The most effective and efficient method of aBME is unclear. Moreover, variability in the frequency and duration of expression was noted. Forster et al. recommended expressing twice daily for 10 min until hospital admission or delivery [13, 21]. In contrast, Lamba and colleagues recommended expressing at least once daily for 5 min [40], Weinel et al. recommended expressing up to 5 min per breast daily or twice daily [33], and Clay et al. recommended expressing three times a day without specifying the length of each expression [43]. The gestational age at which aBME was initiated also varied. Eleven studies recommended expressing between 36 and 37 weeks’ gestation, four studies recommended expressing between 32 and 36 weeks’ gestation, and the remaining recommended expressing earlier or did not state their time of initiation. If the goal is to determine the safety and efficacy of aBME, consensus on the appropriate timing of aBME initiation is required in order to standardize the intervention. Importantly, women with diabetes tend to be induced between 38 and 40 weeks’ gestation, or before if they have poor glycemic control [49, 50]. This should be taken into consideration when establishing a starting point for aBME in this specific population.

The highest-quality study by nature of its RCT design was conducted by Forster et al. [21]. This study included 635 women with gestational or pre-existing diabetes with a singleton, low risk pregnancy. Participants were randomized into an aBME group or a control group who received standard care provided at their local tertiary care facility. The primary outcomes of interest were newborn admission to the NICU and gestational age at birth, which did not differ between the expressing and control groups, repealing the preconceived risks of elevated NICU admission and lower gestational age associated with aBME. This study also reported moderate evidence that infants of women who expressed antenatally were more likely to be exclusively breastfed in the first day of life and during their initial hospital stay (from birth to discharge, or 7 days of age if they had an extended hospital stay). Although data on participant adherence to their assigned intervention were not provided, the research question was clear, the randomization strategy was well defined, the comparison groups were appropriately matched, and the outcome assessors were masked to the allocation of participants to the intervention arms. This study provides a useful framework for the development of protocols for future research. These include the commencement of aBME at 36 weeks’ gestation in mothers with diabetes, assessment of the duration and frequency of antenatal expressing episodes, collection and measurement of colostrum, and lastly, tracking the administration of colostrum to newborns. Where there are many maternal and newborn outcomes related to aBME that may be explored, establishing clear research questions will be essential to advancing knowledge on the safety, efficacy and acceptability of the practice and its effect on measured outcomes.

A multitude of maternal and newborn outcomes following aBME were investigated across the included studies. The most commonly reported outcomes focused on the collection and use of colostrum, breastfeeding status and success at various time points, and outcomes related to newborn health and safety. Documentation of the volumes of colostrum that mothers express, and the amount administered to newborns is important to provide insight into the impact of using expressed breastmilk on newborn health versus with standardized newborn hypoglycemia management protocols which can include enteral feeds of breastmilk, breastmilk substitutes, intrabuccal dextrose gel, or intravenous glucose to stabilize newborn blood glucose levels [51]. Outcomes related to breastfeeding success and status were common among the studies included in this scoping review, however breastfeeding success was often not defined and duration of participant follow-up varied greatly. Three studies reported on time to establishing full lactation [16, 40, 41], and one reported standardized LATCH scores [38]. Definitions used to ascertain ‘time to full lactation’ were unclear however, and likely incongruent with accepted definitions [52]. Although many measures can be used to evaluate breastfeeding success, there is currently no single accepted measurement tool to assess this outcome [53]. Lastly, newborn health and safety outcomes were of particular interest due to recent concerns around preterm birth and increased NICU admission following aBME. Forster et al. evaluated these outcomes in their RCT and did not find any evidence that aBME was unsafe for newborns [21].

Evaluating the receptivity of women to aBME, their feedback on aBME resources and supports, as well as their perceptions and experiences with aBME are essential for informing the reasonable implementation of aBME research studies, clinical recommendations and programming. Among the studies included in this review, data on maternal experiences were captured mostly by interviews (semi-structured and structured) and questionnaires. Findings demonstrated that mothers had a generally positive outlook on aBME. Mothers reported feeling a sense of ownership and confidence with breastfeeding after practicing aBME, however mothers also complained of discomfort and frustration with the process.

Strengths of this scoping review include the use of established frameworks to ensure the unbiased identification and appraisal of relevant studies. The scoping review methodology allowed us to apply a broad research question and iterative search strategy to conduct a comprehensive synthesis of the current literature on aBME. Our methodology was significantly strengthened through our incorporation of a critical appraisal and clinical consultation which allowed us to provide new perspectives to this topic. This review was limited by the fact that we could only include articles published in English. We may also have excluded relevant studies because we were unable to retrieve the full texts at the time of data collection.

## Conclusions

This review demonstrates a lack of high-quality evidence on the effects of aBME on maternal and newborn outcomes. Published studies on the maternal and newborn outcomes of aBME vary widely in their hypotheses and objectives, target populations, interventions, and outcomes of interest, thus hampering comparison across studies. Future work should focus on clear hypothesis driven approaches and provide published or registered protocols outlining the method of breast expression, the timing of the intervention, the storage and use of the colostrum collected, and infant formula supplementation for breastfed infants. Lastly, having well-described populations with appropriate comparison groups is imperative to minimize confounding variables and mediating factors. Given that aBME is thought to be particularly beneficial to pregnant women with diabetes, future studies should consider primarily evaluating outcomes in this population. For researchers conducting trials on mothers with diabetes, willingness to participate and satisfaction with the intervention should be considered as these women often experience other unique burdens in pregnancy such as visits with health specialists and frequent blood glucose monitoring. Our findings provide insight into the many outcomes and experiences that can be considered in the evaluation of aBME and may be useful for informing the development and implementation of future research.

## Supplementary Information

**Additional file 1.** Literature Search Strategy.

**Additional file 2.** Data collection form template.

**Additional file 3.** Detailed critical appraisal of included studies.

## Data Availability

The data analyzed during this scoping review of the literature are publicly available.

## Abbreviations

aBME: Antenatal breastmilk expression
CINAHL: Cumulated Index to Nursing and Allied Health Literature
EBSCOHost: Online research platform
EThOS: E-Theses Online Services
ICU: Intensive care unit
LATCH scores: Latch, Audible swallowing, Type of nipple, Comfort, Hold
MMAT: Mixed Methods Appraisal Tool
NICU: Neonatal intensive care unit
OVID: Ovid is a platform that hosts databases
MEDLINE: Is a database that is hosted on the OVID platform
RCT: Randomized controlled trial
SCBU: Special care baby unit
SCN: Special care nursery
