# Supplementary material for: Evaluating antenatal breastmilk expression outcomes: a scoping review
Source: Int Breastfeed J. 2021 Mar 12;16:25. doi: 10.1186/s13006-021-00371-7 (PMC7971107; doi:10.1186/s13006-021-00371-7)
Supplement: Supplementary file 1 — Additional file 1. Literature Search Strategy. [file 13006_2021_371_MOESM1_ESM.docx]

**Additional file 1: Literature search strategy**

*(Pregnancy, pregnant women, pregnan*, gestational*, maternal*, maternity*) and aBME (breast milk expression or ((antenatal* or before birth* or prenatal) adj5 (breastmilk* or breast milk* or colostrum or (milk adj2 express*))).*

OVID final search strategy:

1. exp Pregnancy/

2. Pregnant Women/

3. exp Diabetes, Gestational/

4. (pregnan* or gestational* or maternal* or maternity).ti,ab,kw.

5. 1 or 2 or 3 or 4

6. breast milk expression/

7. ((antenatal* or before birth* or prenatal) adj5 (breastmilk* or breast milk* or (milk adj2 express*))).ti,ab,kw.

8. 6 or 7

9. 5 and 8
