# Supplementary material for: Evaluating antenatal breastmilk expression outcomes: a scoping review
Source: Int Breastfeed J. 2021 Mar 12;16:25. doi: 10.1186/s13006-021-00371-7 (PMC7971107; doi:10.1186/s13006-021-00371-7)
Supplement: Supplementary file 2 — Additional file 2. Data collection form template. [file 13006_2021_371_MOESM2_ESM.docx]

**Additional file 2: Data collection form template**

| ***Bibliometric Details*** |
| --- |
| Study Title |
| Study Authors |
| Year of Publication |
| Study Journal |
| Study citation details (volume(issue):pages. doi) |
| ***Study Details*** |
| **Type of study** |
| **Country of origin** |
| **Objective(s)** |
| **Setting** |
| **Study Population(s)** |
| *Sample size* |
| *Inclusion criteria* |
| *Exclusion criteria* |
| **Study period** |
| **Outcome measure(s)** |
| ***Methods*** |
| **Advice/support on antenatal BME +/- collection and storage of colostrum** |
| **Data collection method** |
| **Randomization/masking strategy (RCT only)** |
| **Intervention** |
| *Timing of intervention* |
| *Method of aBME* |
| *Instructed frequency of aBME* |
| *Collection and storage of breastmilk* |
| **Comparator** |
| ***Results*** |
| **Main findings** |
| **Maternal age** |
| **High risk subgroups** |
| **Parity** |
| **BMI** |
| **Maternal outcomes** |
| *Antenatal expressing outcomes* |
| *Breastfeeding outcomes* |
| *Maternal experiences* |
| **Infant outcomes** |
| **Reasons for receiving artificial milk** |
| **Cost effectiveness** |
| **Potential references** |
| **Other comments** |
