# Supplementary material for: Evaluating antenatal breastmilk expression outcomes: a scoping review
Source: Int Breastfeed J. 2021 Mar 12;16:25. doi: 10.1186/s13006-021-00371-7 (PMC7971107; doi:10.1186/s13006-021-00371-7)
Supplement: Supplementary file 3 — Additional file 3. Detailed critical appraisal of included studies. [file 13006_2021_371_MOESM3_ESM.docx]

**Additional file 3: Detailed critical appraisal of included studies**

| **Methodological quality criteria** | **Assessment** | **Comments** |
| --- | --- | --- |
| **Blaikley et al. (1953) (quantitative non-randomized)** |  |  |
| S1: Are there clear research questions? | Yes | Repeat experiment of Waller (1946). |
| S2: Do the collected data allow to address the research questions? | No | Come outcome measures not reliable and confounding factors uncontrolled. |
| 3.1 Are the participants representative of the target population? | Yes | 200 primiparous women attending an antenatal clinic and living within 3 miles of the hospital at which the study was conducted. |
| 3.2 Are measurements appropriate regarding both the outcome and intervention (or exposure)? | No | Outcomes of interest were not all stated in the methodology. ‘Fully BF’ outcome was not well defined. |
| 3.3 Are there complete outcome data? | Yes | >90% participant retention for both study groups. |
| 3.4 Are the confounders accounted for in the design and analysis? | No | Sample limited based on parity, maternal age, and gestational age at delivery. Not adjusted for any other confounders |
| 3.5 During the study period, is the intervention administered (or exposure occurred) as intended? | Can’t tell | Unclear if there were differences in care/instruction received by women who delivered in their homes and were see instead by district midwives. |
| **Brisbane et al. (2015) (qualitative study)** |  |  |
| S1: Are there clear research questions? | Yes | Explore the experiences and BF outcomes of a group of mothers who expressed colostrum in the antenatal period |
| S2: Do the collected data allow to address the research questions? | Yes | Collected seven response themes that described women’s experiences with the antenatal expression of colostrum. |
| 1.1 Is the qualitative approach appropriate to answer the research question? | Yes | In-depth interviews were conducted with 12 women who attended a lactation clinic. |
| 1.2 Are the qualitative data collection methods adequate to address the research question? | Can’t tell | Unclear if they used a validated measure of maternal confidence or if they used open-ended questions. |
| 1.3 Are the findings adequately derived from the data? | Can’t tell | Qualitative content analysis was used; however, the approach was inferred as it was not explicitly stated. |
| 1.4 Is the interpretation of results sufficiently substantiated by data? | Yes | Themes were well described and supported by quotations. |
| 1.5 Is there coherence between qualitative data sources, collection, analysis, and interpretation? | Yes | Semi-structured interviews lead to the collection of maternal experiences which provided accurate data that was interpreted appropriately. |
| **Brown et al. (1975) (quantitative randomized control trial)** |  |  |
| S1: Are there clear research questions? | Yes | Evaluate three specific types of prenatal breast preparation in reducing objective and subjective signs and symptoms of nipple problems that may lead to ineffective nursing. |
| S2: Do the collected data allow to address the research questions? | No | Unreliable measures, uncontrolled factors postpartum that could influence mom’s BF experience and nipple pain. |
| 2.1 Is randomization appropriately performed? | No | Women were randomly assigned to one of the 3 interventions. The randomization procedure for group assignment is not described. The breast to be prepared was chosen randomly by a coin toss. |
| 2.2 Are the groups comparable at baseline? | Can’t tell | No group data presented. |
| 2.3 Are there complete outcome data? | Can’t tell | The authors do not provide specific outcome data, but instead summarize the data very briefly. |
| 2.4 Are outcome assessors blinded to the intervention provided? | Yes | Women were asked to not reveal 'which breast' was selected to the nurse who would inspect her breast postnatally. Women's bias to breast selection not blinded. |
| 2.5 Did the participants adhere to the assigned intervention? | Yes | Participants who reported postnatally that they did not adhere to the regime of the group they were assigned were discarded from the sample. |
| **Casey et al. (2019) (quantitative non-randomized)** |  |  |
| S1: Are there clear research questions? | Yes | Compare rates of neonatal hypoglycemia in babies born to mothers who express and store antenatal colostrum to babies born to mothers who do not. |
| S2: Do the collected data allow to address the research questions? | Yes | Research question was answered with the collected data. |
| 3.1 Are the participants representative of the target population? | Yes | Inclusion and exclusion criteria were clear. Participants were consecutively sampled from women in antenatal diabetes register. |
| 3.2 Are measurements appropriate regarding both the outcome and intervention (or exposure)? | No | Although variables were well defined, it is unclear how certain measures were taken. |
| 3.3 Are there complete outcome data? | Yes | Very little missing data. |
| 3.4 Are the confounders accounted for in the design and analysis? | No | Number of episodes of aBME, and timing of aBME not accounted for. Unclear reason for exclusion of preterm births. |
| 3.5 During the study period, is the intervention administered (or exposure occurred) as intended? | Can’t tell | Unsure if expressed breastmilk was used for prevention or treatment of hypoglycemia. |
| **Casey et al. (2019) (qualitative study)** |  |  |
| S1: Are there clear research questions? | Yes | Explore the perspectives and experiences of women who have had diabetes in pregnancy and were encouraged to collect and store colostrum in the antenatal period. |
| S2: Do the collected data allow to address the research questions? | Yes | Collected data appropriately answered the research question. |
| 1.1 Is the qualitative approach appropriate to answer the research question? | Yes | Data was analyzed with thematic analysis. |
| 1.2 Are the qualitative data collection methods adequate to address the research question? | Yes | Face-to-face, semi-structured interviews analyzed with purposive sampling and thematic analysis. |
| 1.3 Are the findings adequately derived from the data? | Yes | Methods to derive the findings were robust and thoroughly described. |
| 1.4 Is the interpretation of results sufficiently substantiated by data? | Yes | Extensive thematic analysis with themes and sub-themes report. Illustrative quotes provided. |
| 1.5 Is there coherence between qualitative data sources, collection, analysis, and interpretation? | Yes | Qualitative data collected and analyzed provided insight into women’s experiences and perspectives about aBME |
| **Clay (2005) (qualitative study)** |  |  |
| S1: Are there clear research questions? | No | Described an example of multidisciplinary care in an antenatal diabetes clinic but did not outline a specific research question or objective. |
| S2: Do the collected data allow to address the research questions? | No | Explained that a multidisciplinary team can work effectively to support colostrum harvesting; did not answer any research questions. |
| 1.1 Is the qualitative approach appropriate to answer the research question? | Can’t tell | No research question(s). |
| 1.2 Are the qualitative data collection methods adequate to address the research question? | No | Describes cases but not data collection. There is no research question. |
| 1.3 Are the findings adequately derived from the data? | Can’t tell | No formal data collection or analysis. |
| 1.4 Is the interpretation of results sufficiently substantiated by data? | No | Study results not generalizable or transferrable. Conclusion statement is not supported by any data. |
| 1.5 Is there coherence between qualitative data sources, collection, analysis, and interpretation? | No | There was no clear research question, limited to one case description with a conclusion that team work is possible to support colostrum harvesting but no data collected to validate this claim. |
| **Demirci et al. (2018) (quantitative descriptive)** |  |  |
| S1: Are there clear research questions? | Yes | Report on maternal experiences and breastfeeding outcomes in mothers with a hypertensive disorder of pregnancy who engaged in aBME. |
| S2: Do the collected data allow to address the research questions? | No | Authors state 4 cases that illustrate a potential association between hypertensive disorders of pregnancy and suboptimal BF outcomes. |
| 4.1 Is the sampling strategy relevant to address the research question? | Yes | Targeted selection of cases of interest from a larger study. |
| 4.2 Is the sample representative of the target population? | Yes | Cases selected because they are examples of target population and issue of interest - hypertensive disorders of pregnancy. |
| 4.3 Are the measurements appropriate? | Yes | Data variables clearly defined and appropriate to the study’s objectives. |
| 4.4 Is the risk of nonresponse bias low? | Yes | Cases were purposefully selected. |
| 4.5 Is the statistical analysis appropriate to answer the research question? | Can’t tell | Only descriptive analyses conducted; but not clearly stated or justified |
| **Demirci et al. (2019) (qualitative study)** |  |  |
| S1: Are there clear research questions? | Yes | Examine the experiences of first-time mothers in the United States who participated in a pilot study of aBME. |
| S2: Do the collected data allow to address the research questions? | Yes | Collected data provides sufficient information to answer the research question. |
| 1.1 Is the qualitative approach appropriate to answer the research question? | Yes | Qualitative descriptive approach was appropriate for the given research question. |
| 1.2 Are the qualitative data collection methods adequate to address the research question? | Yes | Interviews were conducted using a semi-structured script. The interview guide was modified to establish consensus and divergence in emerging themes. |
| 1.3 Are the findings adequately derived from the data? | Yes | Interviews were coded by two authors. A third author refined codes in terms of their properties and dimensions and grouped them into categories reflective of themes and subthemes using analytic techniques. |
| 1.4 Is the interpretation of results sufficiently substantiated by data? | Yes | Quotes provided throughout to support findings. |
| 1.5 Is there coherence between qualitative data sources, collection, analysis, and interpretation? | Yes | Qualitative data collected and analyzed provided insight into women’s experiences with aBME |
| **Fair et al. (2018) (qualitative study)** |  |  |
| S1: Are there clear research questions? | Yes | Assess women’s knowledge, practices, and opinions of aBME as well as any differences within the overweight and obese subgroups |
| S2: Do the collected data allow to address the research questions? | Yes | The results provided information on the knowledge, opinions, and practices of aBME. |
| 1.1 Is the qualitative approach appropriate to answer the research question? | Yes | Qualitative data is appropriate to explore women's opinions about aBME. |
| 1.2 Are the qualitative data collection methods adequate to address the research question? | No | A questionnaire was used with a mixture of question types including free test and fixed response options. Focus groups or phone interviews would have allowed the researchers to collect more information. |
| 1.3 Are the findings adequately derived from the data? | Yes | Report positive, negative, and uncertain perceptions. |
| 1.4 Is the interpretation of results sufficiently substantiated by data? | Yes | Quotes are provided for each theme. |
| 1.5 Is there coherence between qualitative data sources, collection, analysis, and interpretation? | Can’t tell | Not clear how many respondents answered the open-ended questions and how representative the responses are of this population. |
| **Forster et al. (2011) (quantitative non-randomized)** |  |  |
| S1: Are there clear research questions? | Yes | Determine the feasibility and begin to assess the safety of aBME with the objective of conducting an RCT in the future. |
| S2: Do the collected data allow to address the research questions? | No | Concluded that the small sample size was not adequate to examine the safety or efficacy of aBME. However, their data demonstrated that it would be feasible and desirable to conduct an RCT with the same intervention for pregnant women with diabetes requiring insulin. |
| 3.1 Are the participants representative of the target population? | Yes | 43 consecutive women meeting well-defined inclusion criteria with pre-existing or gestational diabetes participated in the study. |
| 3.2 Are measurements appropriate regarding both the outcome and intervention (or exposure)? | Yes | Used accepted measurements to obtain their outcomes and implement their intervention. |
| 3.3 Are there complete outcome data? | No | Tables show substantial incomplete data. Only 26/43 maternal expressing diaries were received. Reasons for incomplete data or loss to follow up not explained. |
| 3.4 Are the confounders accounted for in the design and analysis? | No | Authors excluded cases that may present with confounding variables; however, the data was not adjusted or stratified. |
| 3.5 During the study period, is the intervention administered (or exposure occurred) as intended? | Can’t tell | No fidelity measures reported, only 26/43 women returned their expressing diaries. |
| **Forster et al. (2017) (quantitative randomized control trial)** |  |  |
| S1: Are there clear research questions? | Yes | Explore the safety and efficacy of aBME in mothers with diabetes for the mother, fetus, and newborn infant. |
| S2: Do the collected data allow to address the research questions? | Yes | Research question is addressed with the collected data. |
| 2.1 Is randomization appropriately performed? | Yes | Computerized random number generator in block sizes 2 and 4 and participants were stratified by site, parity, and diabetes type. |
| 2.2 Are the groups comparable at baseline? | Yes | Groups were similar at baseline based on reported baseline characteristics. |
| 2.3 Are there complete outcome data? | No | 3 women were not included in the outcome data. The cost effectiveness was also not reported. |
| 2.4 Are outcome assessors blinded to the intervention provided? | Yes | Investigators were masked to block size, but masking of caregivers was not possible. |
| 2.5 Did the participants adhere to the assigned intervention? | Can’t tell | The total number of expressing episodes are provided, but info on the number adhering to the actual intervention is not provided. |
| **Ingelman-Sundberg (1958) (Quantitative non-randomized)** |  |  |
| S1: Are there clear research questions? | No | Unclear what advantages are specifically being assessed. |
| S2: Do the collected data allow to address the research questions? | No | Conclusion states that aBME has no value and may increase infections. |
| 3.1 Are the participants representative of the target population? | No | Limited to women in one lying in ward. |
| 3.2 Are measurements appropriate regarding both the outcome and intervention (or exposure)? | No | Outcomes were not defined or justified. Only three outcomes were reported on: ‘full-breastfeeding’, total milk, and mastitis. There is insufficient information on the applied intervention. |
| 3.3 Are there complete outcome data? | Can’t tell | Total number of participants that were available/eligible, participated, dropped out/excluded was not provided. |
| 3.4 Are the confounders accounted for in the design and analysis? | No | Was not addressed. |
| 3.5 During the study period, is the intervention administered (or exposure occurred) as intended? | Can’t tell | Instructions on breast massage and antenatal colostrum expression were given starting at the 20th week of gestation, however no information is given beyond the fact that women ‘personally controlled’ their treatment. |
| **Lamba et al. (2016) (quantitative randomized controlled trial)** |  |  |
| S1: Are there clear research questions? | Yes | Study the effect of aBME at term pregnancy and subsequent effect on postnatal lactation performance. |
| S2: Do the collected data allow to address the research questions? | Yes | Results answered the research question. |
| 2.1 Is randomization appropriately performed? | Can’t tell | Randomization not detailed. |
| 2.2 Are the groups comparable at baseline? | Can’t tell | Baseline data not presented, group characteristics not reported. |
| 2.3 Are there complete outcome data? | Yes | Complete outcome data provided for each group. |
| 2.4 Are outcome assessors blinded to the intervention provided? | Can’t tell | No description of blinding provided. |
| 2.5 Did the participants adhere to the assigned intervention? | Can’t tell | No fidelity measures reported. |
| **O’Sullivan et al. (2019) (qualitative study)** |  |  |
| S1: Are there clear research questions? | Yes | Explore the perspectives and experiences of women who have had diabetes in pregnancy and were encouraged to collect and store colostrum in the antenatal period. |
| S2: Do the collected data allow to address the research questions? | Yes | Data collected permitted authors to appropriately address their research question. |
| 1.1 Is the qualitative approach appropriate to answer the research question? | Can’t tell | Little information was given on the qualitative approach used. |
| 1.2 Are the qualitative data collection methods adequate to address the research question? | No | Limited to questionnaire responses (open text); not interviews/focus groups. |
| 1.3 Are the findings adequately derived from the data? | Can’t tell | Data analysis not detailed; however, quotes were provided throughout to support findings. |
| 1.4 Is the interpretation of results sufficiently substantiated by data? | Yes | Illustrative quotes were provided throughout |
| 1.5 Is there coherence between qualitative data sources, collection, analysis, and interpretation? | Yes | Qualitative data collected and analyzed provided insight into women’s experiences and perspectives about aBME? |
| **Rietveld (2011) (mixed methods)** |  |  |
| S1: Are there clear research questions? | Yes | Aim of study is (1) find out if women with type 1, type 2, or gestational diabetes can achieve antenatal colostrum harvesting and banking; and (2) assess feasibility of mothers and core midwifery staff using banked colostrum in the hospital setting as part of care for babies with hypoglycemia. |
| S2: Do the collected data allow to address the research questions? | Yes | Data collected answers the two given research questions. |
| 5.1 Is there an adequate rationale for using a mixed methods design to address the research question? | Yes | Multiple methods were used. Qualitative methods were only used for data collection to answer specific questions about maternal experience and feasibility. Quantitative methods were used to obtain the rest of the data. |
| 5.2 Are the different components of the study effectively integrated to answer the research question? | No | Qualitative assessment was not done throughout the study, it was only conducted at the end and is therefore not truly integrated. |
| 5.3 Are the outputs of the integration of qualitative and quantitative components adequately interpreted? | No | Minimal interpretation of the qualitative findings relative to the quantitative component. |
| 5.4 Are divergences and inconsistencies between quantitative and qualitative results adequately addressed? | Yes | No divergence between quantitative and qualitative results noted. |
| 5.5 Do the different components of the study adhere to the quality criteria of each of the methods involved? | No | When assessed for quantitative descriptive and qualitative studies criteria, the quality criteria were not met or were unclear. |
| **Singh et al (2009) (Quantitative randomized control trials)** |  |  |
| S1: Are there clear research questions? | Yes | Determine the effect of aBME at term in reducing BF failures compared to conventional BF methods. |
| S2: Do the collected data allow to address the research questions? | Yes | Data collected on BF initiation, BM flow after initiation, BF success/failure, time to establish full BF, newborn satisfaction. Determined that daily aBME after 37 weeks’ gestation reduced time to full lactation and decreased BF failures. |
| 2.1 Is randomization appropriately performed? | Can’t tell | Randomization strategy not detailed. |
| 2.2 Are the groups comparable at baseline? | Can’t tell | Unable to determine if groups were comparable for anything other than parity. |
| 2.3 Are there complete outcome data? | Yes | Data available on all participants. |
| 2.4 Are outcome assessors blinded to the intervention provided? | Can’t tell | Blinding was not detailed in the methods. |
| 2.5 Did the participants adhere to the assigned intervention? | Can’t tell | Not documented. |
| **Soltani et al. (2012) (Quantitative non-randomized)** |  |  |
| S1: Are there clear research questions? | Yes | Investigate the pattern of aBME uptake and its relationship to birth outcomes in women with diabetes in pregnancy. |
| S2: Do the collected data allow to address the research questions? | Yes | Determined that ½ of women who were told to express, did so. They found an association between aBME and lower gestational age at birth as well as increased SCBU admission compared to the non-expressing group. |
| 3.1 Are the participants representative of the target population? | Yes | Clear target population and appropriate inclusion and exclusion criteria. |
| 3.2 Are measurements appropriate regarding both the outcome and intervention (or exposure)? | Yes | Measures collected were well described in the methods. |
| 3.3 Are there complete outcome data? | Yes | Majority of women who returned a survey completed it in full. |
| 3.4 Are the confounders accounted for in the design and analysis? | No | No adjustments made in data analysis for potential confounders. |
| 3.5 During the study period, is the intervention administered (or exposure occurred) as intended? | Can’t tell | Intervention was not explicitly defined in the methodology. |
| **Uikey et al. (2017) (quantitative randomized controlled trial)** |  |  |
| S1: Are there clear research questions? | Yes | Study the effect of aBME at term in improving lactational performance as compared to a control group. |
| S2: Do the collected data allow to address the research questions? | Yes | Data collected answered the research question. |
| 2.1 Is randomization appropriately performed? | Can’t tell | Randomization is not detailed in the methods. |
| 2.2 Are the groups comparable at baseline? | Can’t tell | There is no baseline data presented in the results. |
| 2.3 Are there complete outcome data? | Yes | All outcome data appear to be provided. |
| 2.4 Are outcome assessors blinded to the intervention provided? | Can’t tell | There is no description of blinding to data collection in the methods. |
| 2.5 Did the participants adhere to the assigned intervention? | Can’t tell | There were no fidelity measures described. |
| **Waller (1946) Interventional study (quantitative non-randomized)** |  |  |
| S1: Are there clear research questions? | Yes | Explore the impact of teaching aBME in the last weeks of pregnancy on outflow of milk. |
| S2: Do the collected data allow to address the research questions? | No | Outcome measures are not reliable and confounding factors are not controlled. |
| 3.1 Are the participants representative of the target population? | Can’t tell | Authors did not provide a clear description of the inclusion/exclusion criteria and why certain eligible individuals may have chosen not to participate. |
| 3.2 Are measurements appropriate regarding both the outcome and intervention (or exposure)? | No | Unclear as to how many outcomes were measured or defined (ex. Freedom of milk outflow, incidence of overfilling/engorgement, incidence of nipple trauma) |
| 3.3 Are there complete outcome data? | Yes | No dropouts or losses to follow up are reported implying full data set was obtained from all participants. |
| 3.4 Are the confounders accounted for in the design and analysis? | No | Parity was controlled for by excluding multiparous women, however no other confounders were accounted for. |
| 3.5 During the study period, is the intervention administered (or exposure occurred) as intended? | Can’t tell | Unclear if the control group were taught to express breastmilk antenatally as well. No measure of fidelity detailed. |
| **Weinel et al. (2019) (quantitative descriptive)** |  |  |
| S1: Are there clear research questions? | No | No true research questions. Objective was to support mothers with pre-existing or gestational diabetes, and who were on more than 20 units of insulin per day, to express colostrum in the antenatal period at 36 weeks' gestation and to promote breastfeeding. |
| S2: Do the collected data allow to address the research questions? | No | No research question or outcome measures. |
| 4.1 Is the sampling strategy relevant to address the research question? | Can’t tell | Sampling strategy not detailed. |
| 4.2 Is the sample representative of the target population? | Yes | Purposeful sample of women from Diabetic antenatal care education clinic. |
| 4.3 Are the measurements appropriate? | No | Variables are not clearly defined; outcome measures not clearly defined or justified. |
| 4.4 Is the risk of nonresponse bias low? | No | 383 women approached 207 actually attended the clinic 141 were re-contacted to assess breastfeeding postpartum. Reasons for drop-out/no response are well detailed |
| 4.5 Is the statistical analysis appropriate to answer the research question? | Can’t tell | No statistical analysis detailed. |

BF, breastfeeding; BM, breastmilk
